# Vitamin C Transporters, Recycling and the Bystander Effect in the Nervous System: SVCT2 versus Gluts

**DOI:** 10.4172/2157-7633.1000209

**Published:** 2014-05-19

**Authors:** Francisco Nualart, Lauren Mack, Andrea García, Pedro Cisternas, Ernesto R. Bongarzone, Marjet Heitzer, Nery Jara, Fernando Martínez, Luciano Ferrada, Francisca Espinoza, Victor Baeza, Katterine Salazar

**Affiliations:** 1 Center for Advanced Microscopy CMA BIO-BIO, Neurobiology and Stem cell Laboratory, Concepcion University, Chile; 2 Department of Anatomy and Cell Biology, College of Medicine, University of Illinois Chicago, USA

**Keywords:** Vitamin C: SVCT2: GLUTs: Bystander effect, Tanycytesl, Astrocytes, Stem cells, Neurons, Nervous System, Recycling, Ascorbic acid, Dehydroascorbic acid

## Abstract

Vitamin C is an essential micronutrient in the human diet; its deficiency leads to a number of symptoms and ultimately death. After entry into cells within the central nervous system (CNS) through sodium vitamin C transporters (SVCTs) and facilitative glucose transporters (GLUTs), vitamin C functions as a neuromodulator, enzymatic cofactor, and reactive oxygen species (ROS) scavenger; it also stimulates differentiation. In this review, we will compare the molecular and structural aspects of vitamin C and glucose transporters and their expression in endothelial or choroid plexus cells, which form part of the blood-brain barrier and blood-cerebrospinal fluid (CSF) barrier, respectively. Additionally, we will describe SVCT and GLUT expression in different cells of the brain as well as SVCT2 distribution in tanycytes and astrocytes of the hypothalamic region. Finally, we will describe vitamin C recycling in the brain, which is mediated by a metabolic interaction between astrocytes and neurons, and the role of the “bystander effect” in the recycling mechanism of vitamin C in both normal and pathological conditions.

## Introduction

**Figure 1:**
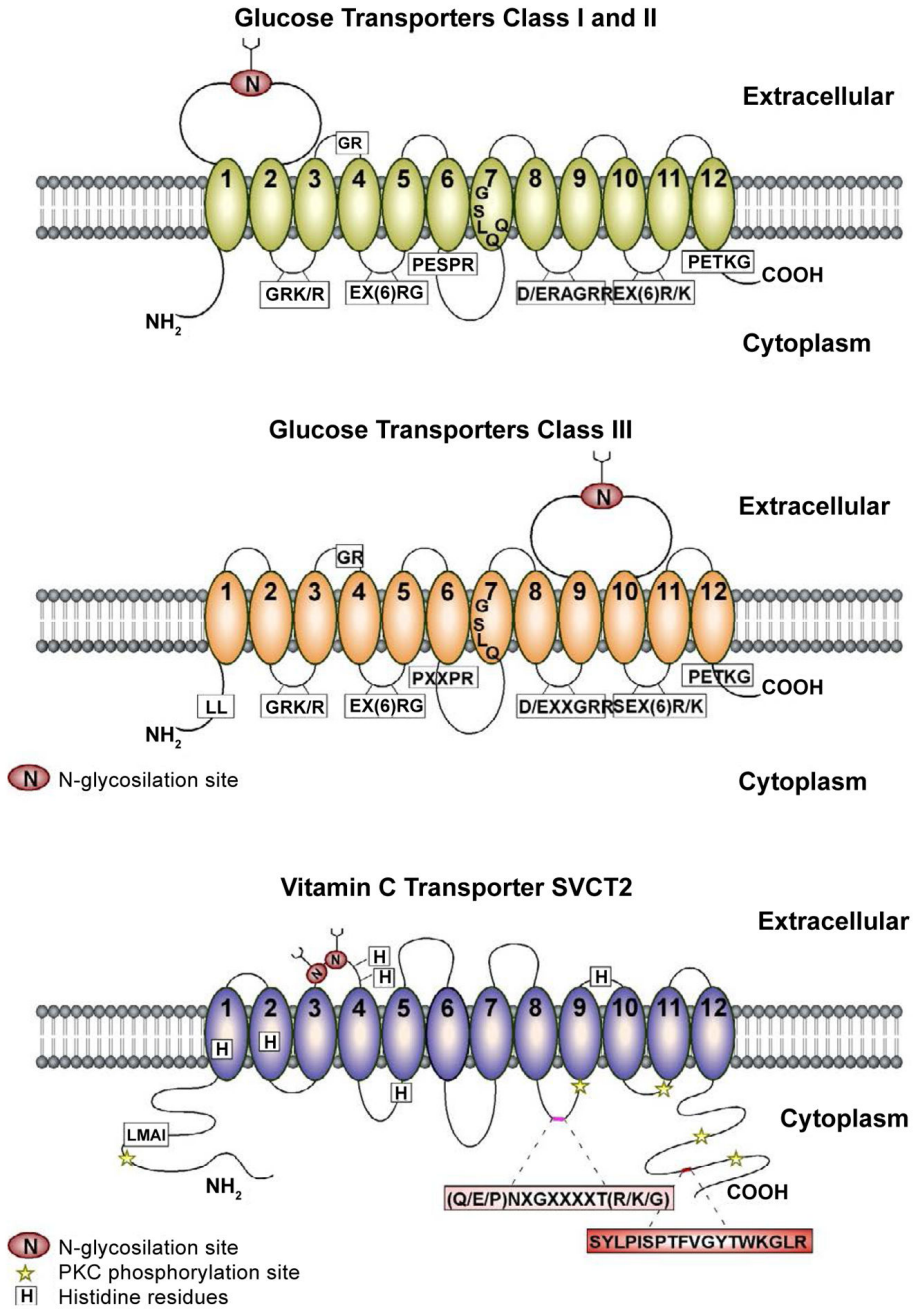
Comparative membrane topologies of Class I, II and III glucose transporters and the vitamin C transporter, SVCT2 Class I, II and III glucose transporters as well as SVCT2 have 12 putative transmembrane domains (TM; numbers 1–12) base on prediction algorithms for transmembrane topology. **A,B.** The signature sequences conserved between classes I and II (A) and class III (B) glucose transporters are shown in boxes. Differences between the position of the large extracellular loop containing the N-glycosylation site, the proline-containing motif between TM6 and TM7, and the presence of a dileucine motif in the amino-terminal tail of class III glucose transporters (except for GLUT10) have been noted. **C.** The consensus sites for N-glycosylation and protein kinase C phosphorylation are shown. Important sequences for SVCT2 function and sorting include five histidine residues (H109, H203, H206, H269 and H413), the signature motif ((Q/E/P)NXGXXXXT(R/K/G)), a basolateral targeting sequence (LMAI) and a C-termini sequence for cell surface targeting, membrane incorporation, and retention (SYLPISPTFVGYTWKGLR) [105–107].

Vitamin C is a micronutrient essential for normal metabolic function; its deficiency in the human diet results in scurvy that is characterized by bleeding gums, impaired wound healing, petechiae, perifollicular hemorrhage, anemia, fatigue, depression, and ultimately death [1]. In the majority of mammals, vitamin C is synthesized in the liver; however, humans, other primates, and guinea pigs have lost their capacity to synthesize vitamin C due to the presence of a non-functional *L-gulono-gamma-lactone oxidase* gene, which is necessary for the last step in ascorbic acid (AA) biosynthesis [2]. In the blood, vitamin C levels reach up to 50 μM with most in its reduced form, AA, and only 5–10% in its oxidized form, dehydroascorbic acid (DHA). Independent of the capacity to synthesize vitamin C, efficient incorporation into the cells is crucial. AA is actively incorporated into the cytoplasmic membrane by sodium vitamin C transporters (SVCTs), and DHA uptake is mediated by facilitative glucose transporters (GLUTs) [3,4]. Specifically, GLUT1 and GLUT3 are mainly responsible for DHA uptake by cells of the central nervous system (CNS; Figure 1) [5,6].

In the brain, an interaction between astrocytes and neurons has been proposed to mediate AA recycling that is crucial for the maintenance of normal brain AA concentrations required to fulfill different functions inside the CNS (e.g., antioxidant protection [7–11], catecholamine biosynthesis [12], peptide amidation [13], myelin formation [14], enhancement of synaptic activity [15], protection against glutamate toxicity [16] and modulation of precursor cell proliferation and differentiation [17,18]). Neurons incorporate AA where it is converted to DHA, which modifies neuronal function (e.g., modifying the glycolysis and pentose-phosphate pathways) [19]. These metabolic changes stimulated by AA, and its intracellular oxidation, increases glia-neuron metabolic coupling, inducing lactate uptake by neurons and astrocyte DHA recycling [20,21]. In pathological conditions where concentrations of nitric oxide synthase (NOS) are increased, these recycling mechanisms may collapse, inducing neuronal toxicity.

Defining the molecular and physiological mechanisms of vitamin C recycling in the CNS and the differential expression of SVCT2 and GLUTs in neurons and astrocytes in normal conditions may illustrate their possible roles in complex pathologies, such as ischemic stroke, and Alzheimer’s or Huntington’s diseases. In this context, the administration of DHA to animals with experimental cerebral stroke has been suggested to reduce neurological deficit and mortality [22]. However, Cisternas et al. [19] have demonstrated that high intracellular DHA concentration inhibits neuronal glycolytic activity, increases the pentose-phosphate pathway and decreases reduced glutathione levels. Therefore, the effects associated with the administration of DHA to the brain should be studied in detail before being used in the treatment of different brain diseases.

Recently, abnormal AA flux from astrocytes to neurons in brain slices from R6/2 Huntington’s disease mice was observed [23]. Additionally, in STHdhQ neurons derived from knock-in mice expressing mutant Huntington, SVCT2 fails to reach the plasma membrane in response to increased AA concentration. Furthermore, *in vitro* and animal studies showed that AA improves the clinical and pathological phenotype of a mouse model of Chercot-Marie-tooth disease 1A (CMT1A) [24,25], which led to initiation of various clinical trials examining AA administration in CMT1A. However, none of these trials showed a significant benefit of AA in the treatment of CMT1A patients [26–28]. The lack of effectiveness in clinical studies was difficult to interpret because there were no studies assessing SVCT2 expression in Schwann cells and peripheral nerves, which was only recently obtained by [29].

These recent studies have opened new concepts concerning the biology of vitamin C and SVCT2 and GLUT expression and function in brain pathologies. In this review, the regulation of vitamin C entry and homeostasis within the CNS will be examined. We will describe the mechanisms of AA recycling between the neuron and astrocytes in detail, discussing whether the brain can implement the “bystander effect” [20,30] through the active SVCTs and GLUTs expressed in these cells.

## Cloning of SVCT2 and Protein Structure

Rat SVCT1 cDNA was initially identified after screening a rat kidney cDNA library for Na+-dependent L-AA transport in functional expression assays of RNA-injected *Xenopus* oocytes. Subsequent polymerase chain reaction (PCR)-based screening yielded a similar cDNA isolated from rat brain, rat SVCT2, which had 65% homology to rat SVCT1 [31]. This study revealed a new family of proteins, the sodium-dependent AA transporter family (SLC23), which contains SVCT1, SVCT2, and an orphan member, SVCT3. The latter was cloned from mouse yolk sac [32], and subsequent studies showed that SVCT3 is predominantly expressed in the kidney. However, there is no functional data for this transporter at present; therefore, it remains unknown whether SVCT3 is an AA or nucleobase transporter [33]. In rats, an additional isoform exists, SVCT4, which is called SLC23A4; it is a nucleobase transporter predominantly expressed in the small intestine. However, in humans, the *SLC23A4* gene is a pseudogene [33]. Human SVCT1 and SVCT2 were subsequently cloned from cDNA libraries of placental choriocarcinoma cells, Caco2 colon carcinoma cells, kidney and human brain [31,34–37]. These transporters are encoded by the *SLC23A1* and *SLC23A2* genes and were previously described as nucleobase transporters YSPL3 and YSPL2, respectively [38]. The mechanism by which SVCT1 and SVCT2 mediate AA transport was characterized after functional expression in *Xenopus* oocytes and transfection into mammalian cells followed by the monitoring of the incorporation of ^14^C-labeled AA. These studies revealed a high affinity and stereospecificity for L-AA, but not DHA, 2-phosphate AA, 2-sulfate AA, L-gluco-γ-lactate and glucose [31,37]. In addition, AA uptake was dependent on Na+ and was inhibited after it was replaced with K+ or Li+ [34,36]. AA uptake by SVCT2-expressing neurons isolated from the brain and cerebellar cortex and HN33.11 and neuroblastoma cell lines was inhibited by the flavonoids, quercetin and phloretin, while no inhibitory effect was seen with the addition of known GLUT inhibitors, cytochalasin B (CytB) or cytochalasin E [39].

The *human SVCT1* and *SVCT2* genes code for proteins of 598 and 650 amino acids, respectively; hydropathy analyses estimate that these transporters possess 12 transmembrane domains with cytoplasmic amino and carboxy termini (Figure 1). Whereas SVCT1 is expressed in epithelial cells of kidney, intestine, and liver, SVCT2 has been detected in a variety of organs and is highly expressed in the brain and eyes [31,34,35,38]. A non-functional splice variant of SVCT1, which possesses four additional amino acids between transmembrane domains 3 and 4, has been discovered in Caco2 colon carcinoma cells as well as in normal human intestine [36]. In addition, SVCT2-short, a nonfunctional splice variant without domains 5 and 6 and part of domain 4, is a truncated protein that is expressed in all adult and fetal tissues analyzed [40]. SVCT2-short strongly inhibits SVCT2 function and partially inhibits SVCT1 in a dominant-negative manner most likely resulting from protein-protein interaction [40]. Thus, SVCT2-short may limit AA transport through these transporters, regulating AA transport to a few tissues despite its widespread mRNA expression.

## Vitamin C entry into the Brain

**Figure 2:**
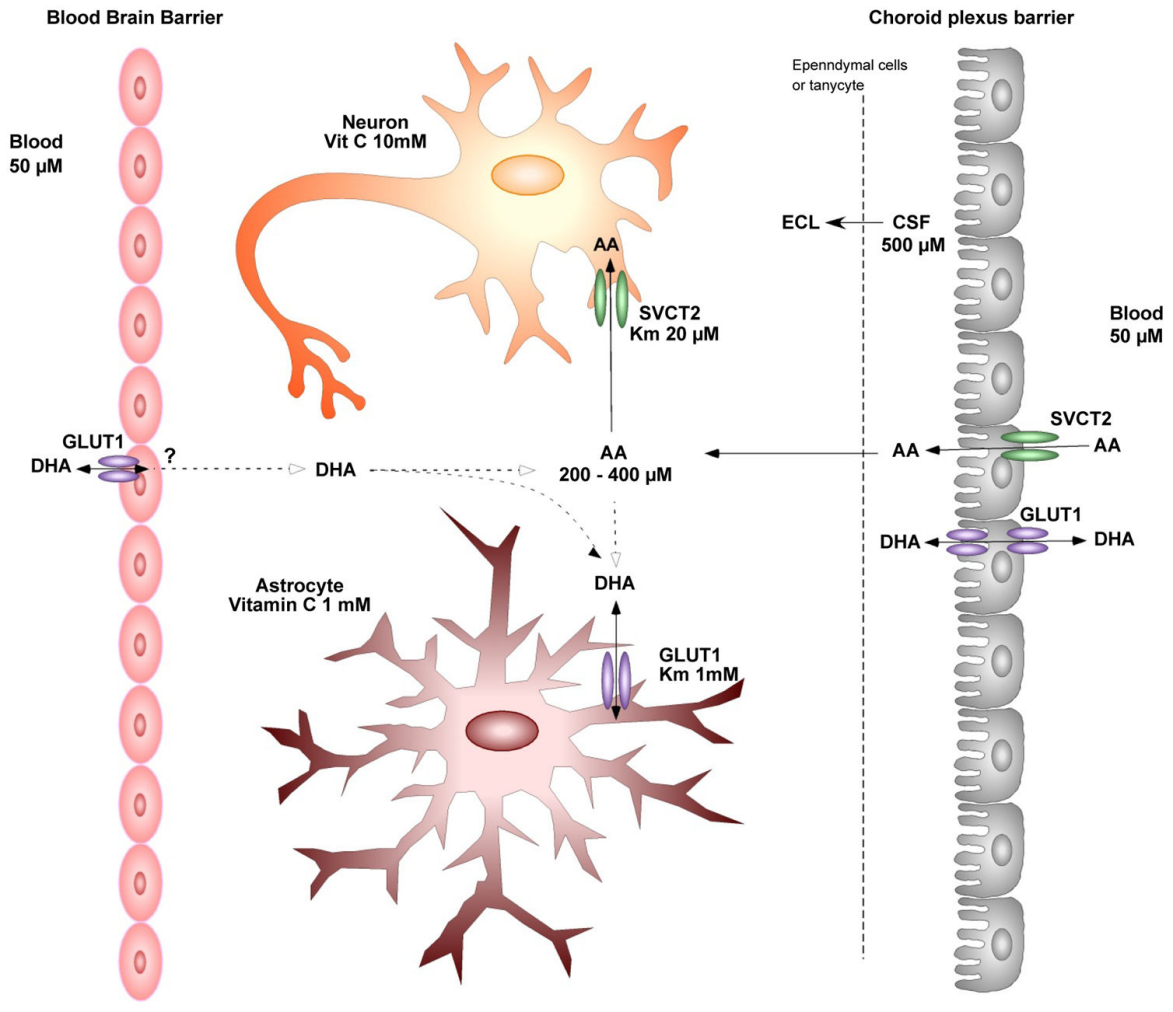
Schematic model of the uptake and compartmentalization of vitamin C in the CNS. Functional activity of astrocytes and tanycytes (hypothalamic stem cells) Vitamin C enters the CNS by SVCT2 present in choroid plexus cells and also probably through GLUT1. The concentration of ascorbate is balanced between cerebrospinal fluid (CSF) and the extracellular fluid (ECF) by diffusion through ependymal cells. Once inside the brain, AA is incorporated by neurons by using SVCT2. SVCT2 is not expressed in astrocytes; thus, it is postulated that astrocytes incorporate DHA through GLUT1. Additionally, it has been postulated that ascorbate also may enter the brain through the blood-brain barrier; however, the mechanism has not been elucidated. (Modified from Rice [62]).

Although it is known that the CNS can maintain ascorbate levels during periods of low and high plasma ascorbate levels [41], the exact mechanism by which AA enters the CNS remains to be elucidated. There are two primary barriers that limit the entry of hydrophilic molecules into the CNS from the: 1) the blood-brain-barrier and 2) the blood-CSF barrier formed by epithelial cells of the choroid plexus [42] (Figure 2). AA entry into the CNS requires passage through one of these barriers aided by either facilitated diffusion or active transport. AA is present in higher concentrations in CSF (200–400 μM) and brain parenchyma than in plasma (30–60 μM) [7,43–45] (Figure 2). This gradient is further increased in neural tissue depending on the cell type and region studied [46,47]. Whole-body radiography at various times after injection with ^14^C-labeled AA demonstrated that it first concentrates in choroid plexus cells and then passes through the CSF into the brain [48]. The requirement of SVCT2 for vitamin C accumulation in the CNS was evident in SVCT2 knock-out mice that have undetectable levels of vitamin C in the brain [49]. Primary cultures of choroid plexus cells also accumulate AA against a concentration gradient [7], which is most likely due to the functional expression of SVCT2 in these cells [31] as observed in choroid plexus epithelium *in vivo* using *in situ* hybridization [31]. Primary choroid plexus cells can also be maintained on permeable membranes thus forming two fluid compartments separated by a cell monolayer simulating the blood-choroid plexus barrier and have been used to study transcellular transport of vitamin C as well as other molecules [50–53]. Angelow et al. [50] reported that AA is transported to the apical side in a concentration-dependent manner in this model with a Km of 67 μM [50,54], which is in agreement with the data derived from uptake measurements with choroid plexus tissue [7,55], SVCT2-cDNA transfected HPRE-cells [34], as well as embryonic mouse neurons [56]. Using this system, the mechanism by which AA is transported was assessed by inhibition studies using phloretin, a facilitative glucose transporter inhibitor that inhibits SVCT, and ouabain, which inhibits the Na-K-ATPase. Both phloretin and ouabain inhibited AA transport and uptake by choroid plexus epithelium in a concentration-dependent manner, demonstrating the importance of the Na-dependent transport of AA in choroid plexus cells [50]. This data also suggests that AA uptake occurs on the basolateral membrane and not the apical membrane, which faces the CSF, although further *in vivo* studies are necessary to confirm this.

One limitation of this system in studying the blood-CSF barrier is the exclusion of the other components of the choroid plexus, such as capillaries and stromal cells. To further study the localization and function of SVCT2 in choroid plexus cells as well as to determine if it is similar in humans, preliminary immunohistochemical analysis of SVCT2 in normal choroid plexus cells was undertaken and revealed that it was principally located within the cytoplasm [57]. However, a strong reaction in the basolateral region was observed in a few regions of the plexus [57]. Due to the difficulties in obtaining normal human choroid plexus cells, further studies utilized resected human choroid plexus papilloma cells obtained from a 16–year-old male [58]. These cells maintain the characteristics of choroid plexus cells as well as the structure of the tissue within the brain. In addition to analyzing SVCT2 localization, the effect of sodium dependence and temperature on these cells was also analyzed. In the absence of sodium, AA incorporation was greatly reduced and was even more significant when performed at 37°C [58]. It must also be mentioned that the mechanisms by which AA enters the brain from choroid plexus cells remains to be elucidated.

AA transport through endothelial cells of the blood-brain-barrier does not occur under normal conditions due to the lack of SVCT2 expression [59]. Historically, the absence or presence of SVCT2 was a highly debated subject; Qiao and May demonstrated by immunohistochemistry and by immunoblotting that SVCT2 protein was expressed in capillaries, but not cortical capillary endothelial cells [59]. However, after seven days in culture, these cells express SVCT2 and transport ascorbate at rates comparable to those in immortalized endothelial cells lines [20]. In one direct *ex vivo* study, Na+-independent AA transport by endothelial cells was demonstrated; however, further studies are needed to verify this postulation [60]. A second pathway for vitamin C to enter the CNS is to pass through the endothelial cells of the blood-brain barrier in its oxidized form, DHA, via the glucose transporter GLUT1 [61]. However, under physiological conditions, vitamin C is primarily present in plasma in its reduced form [62] thus making it unlikely that this is the prominent pathway for vitamin C entry into the CNS. However, it is possible that DHA generation in specific areas of brain blood vessels may stimulate DHA transport within the brain although this mechanism has yet to be demonstrated. In addition to the issue of low DHA concentrations in serum, the mechanism by which DHA uptake by endothelial cells effects AA levels in the brain remains unknown. Although Agus et al. [61] showed *in vivo* that AA levels are increased in total brain after an intravenously injection of DHA into rats, the location within the brain was not investigated [61]. It is possible that the DHA entered endothelial cells through GLUTs and was instantly reduced to AA and therefore sequestered in the endothelial cells without passing to the brain.

## Distribution of SVCT2 in Cells of the Nervous System

### SLC23a1−/− mice

SVCT2-knockout mice (Slc23a1−/−) develop respiratory distress and intraparenchymal brain hemorrhage and die within minutes of birth; however, these abnormalities are limited to the lungs and brain [49]. It appears as though the respiratory failure is caused by a lack of alveolar expansion not a lack of surfactant protein B, suggesting a previously unknown role of SVCT2 in lung maturation [49]. Examination of the brain revealed petechiae and ecchymoses over the convex surface of the brain; however, no bleeding was observed in any other tissues, suggesting that this was not scurvy-related [49]. This was confirmed in that no collagen synthesis modification was observed; 4-hydroxyproline content was the same in knockout mice as compared to wild-type controls [49]. Analysis of AA transport in cultured embryonic fibroblasts from Slc23a1−/− mice revealed less than 5% of normal uptake [49]. The absence of normal AA transport was further confirmed when tissues of newborn Slc23a1−/− mice revealed undetectable levels of AA in the brain, pituitary, adrenals, and pancreas as well as a marked decrease in the liver, kidney, muscles, and blood [49]. Slc23a1+/− heterozygous mice also displayed reduced AA levels in the brain and blood, but not in the liver [49]. Sotiriou et al. [49] also suggest that fetal Slc23a1 is a significant contributor to the maternal-to-fetal transport of AA across the placenta because supplemental AA to pregnant females did not change AA levels in slc23a−/− fetuses while it almost doubled it in wild-type mice [49]. In a more recent study of hippocampal neuronal cultures from SVCT2-deficient mice, stunted neurite outgrowth, less glutamate receptor clustering and reduced spontaneous neuronal activity were observed [15]. Also, the hippocampal cultures showed increased susceptibility to oxidative damage and N-methyl-D-aspartate-induced excitotoxicity [15]. Therefore, SVCT2 maintains intracellular AA levels in neurons, thereby preventing neuronal oxidative damage triggered during normal brain activity.

### Localization of SVCT2 in the distinct cells of the brain

SVCT2 but not SVCT1 is expressed in the fully developed brain [57]. The high expression of SVCT2 in the brain was first observed by Tsukaguchi et al. [31], the group who first cloned the transporter [31]. Immunohistochemistry analyses indicated that the strongest expression of SVCT2 in the brain was in neurons of the cerebral cortex, hippocampus, and Purkinje cells of the cerebellum although other cell types express SVCT2 [57].

Ascorbate content correlates with neuron density, suggesting that neurons are the primary cells which store AA within the CNS [63]. *In situ* hybridization in rat brain first indicated that SVCT2 is localized at high levels in neurons but not glial cells [31,64,65] and is present in both glutamatergic and GABAergic neurons, including glutamatergic pyramidal cells of the hippocampus, glutamatergic granule cells of the cerebellum and GABAergic cerebellar Purkinje cells [57]. Double immunohistochemistry analysis confirmed that most SVCT2-positive cells were neurofilament-positive; anti-neurofilament antibody is an effective marker for the axonal processes of neurons [66]. Within neurons, SVCT2 was primarily observed within the cellular body in the cell membrane and was not present in axonal or dendritic processes [39,57].

Although the presence of SVCT2 in neurons has been well accepted, its expression in astrocytes is less straightforward as conflicting data from *in vivo* or *in vitro* systems have been reported. For example, Wilson et al. [67] proposed that ascorbate is primarily stored within astrocytes in the CNS. However, this data was based on primary cultures of astrocytes that express SVCT2 and have intracellular concentrations of AA that reach up to 7–10mM. Within these cells, cotransporter activity and intracellular AA concentration was increased with the addition of dibutryl cAMP and decreased with hyposomotic cell swelling, low extracellular Na+ and depolarizing levels of extracellular K+ [68]. However, all *in vivo* data reported to data conflict with that observed in primary cultures. *In situ* hybridization in normal and quinolic acid neurotoxin-stimulated astrocytes showed a lack of SVCT2 expression while cultured astrocytes expressed SVCT2 [64]. Thus, SVCT2 expression is most likely an artifact of culturing, which leads to an artificial upregulation; however, further protein localization studies will be necessary to confirm these data. Furthermore, in 2006, Mun and colleagues mapped the protein distribution of SVCT2 within the brain by immunohistochemistry, and observed that SVCT2-positive cells were not positive for glial fibrillary acidic protein (GFAP), an astrocyte cell marker, further demonstrating that SVCT2 is not expressed in astrocytes [57]. Recently, a detailed histological analysis confirmed that most astrocytes were negative for SVCT2 in the different areas of the brain; however, astrocytes in the external area of the entorhinal cortex and marginal zone of the brain showed SVCT2 expression [3]. Experiments using an antisense for SVCT2 (*in situ* hybridization) and anti-GFAP (immunohistochemistry) in the same section confirmed SVCT2 expression in GFAP-positive cells. Additionally, SVCT2 and GFAP double labeling was also detected in the glia limitans, the thin barrier of astrocytes associated with the parenchymal basal lamina surrounding the brain [3]. Thus, SVCT2 is expressed in highly specialized populations of astrocytes.

**Figure 3:**
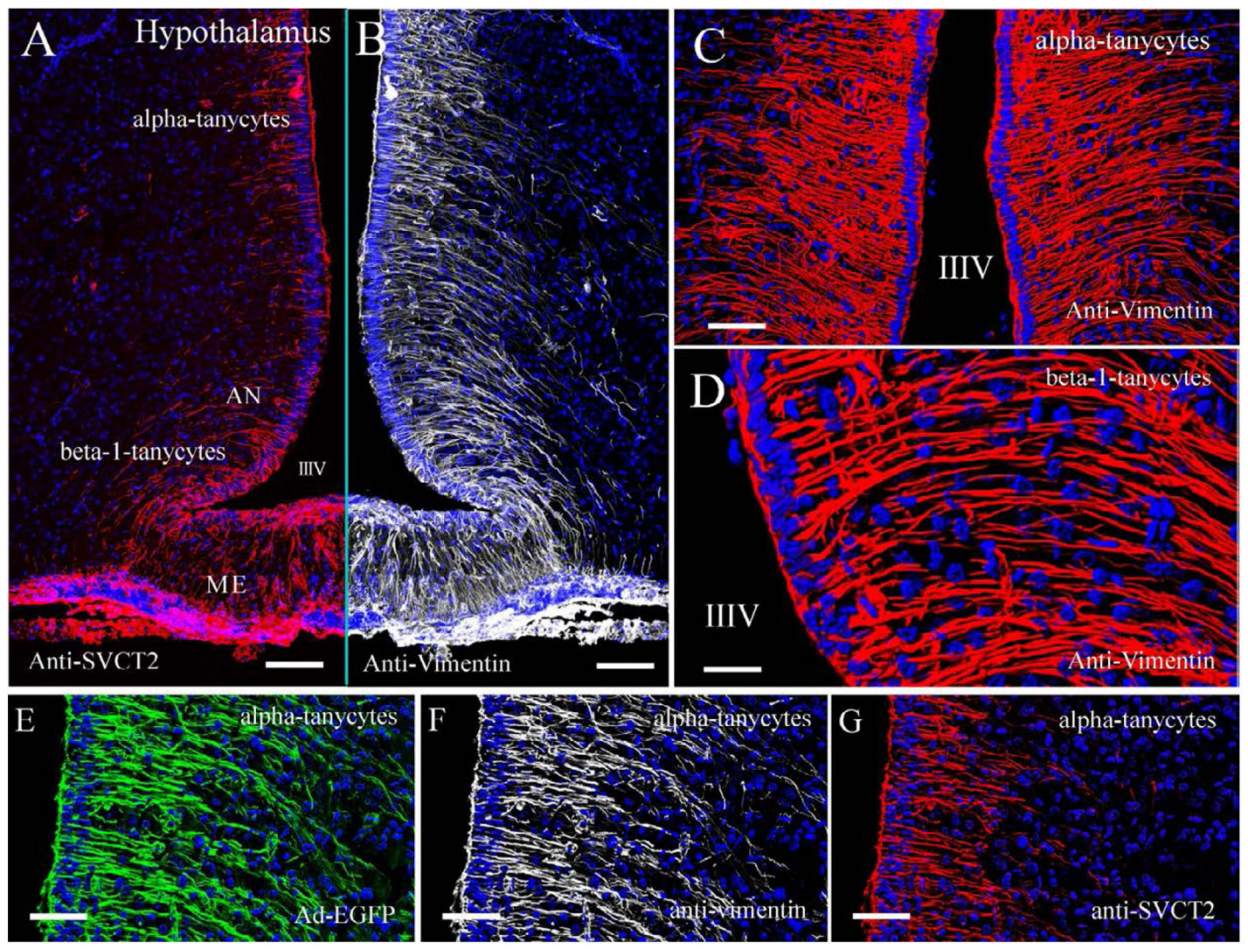
Tanycyte distribution and SVCT2 immunoreaction in hypothalamic cells Tanycytes are specialized hypothalamic glial cells that can be classified as alpha and beta-tanycytes. The tanycytes located in the dorsal walls of the third ventricle are classified as alpha tanycytes, which are involved in neurogenic activity (stem-like cells). Beta-1 tanycytes are located in the lateral lower area of the ventricle and develop elongated cell processes that form a bow through the arcuate nucleus and reach the lateral sulcus of the infundibular region in the lateral hypothalamus. **A.** SVCT2 and **B.** vimentin expression analysis in hypothalamic alpha and beta tanycytes. Detailed tanycyte structure and vimentin immunoreaction are observed in **C and D**. The fluorescence analysis of vimentin (red) and nuclei (Topro in blue) was performed using confocal spectral microscopy (Zeiss 780 equipment), tile scanning, Z-stack imaging and rendering analysis (process of generating an image from a model). **E–G**. Alpha-tanycyte distribution after infection with adenovirus-GFP (E) or fluorescence analysis of vimentin (F) and SVCT2 (G). IIIV, Third ventricle; AN, Arcuate nucleus; ME, Median eminence. Scale bars in A and B, 800 μm; in C and D 400 μm; and in E–G, 200 μm.

**Figure 4:**
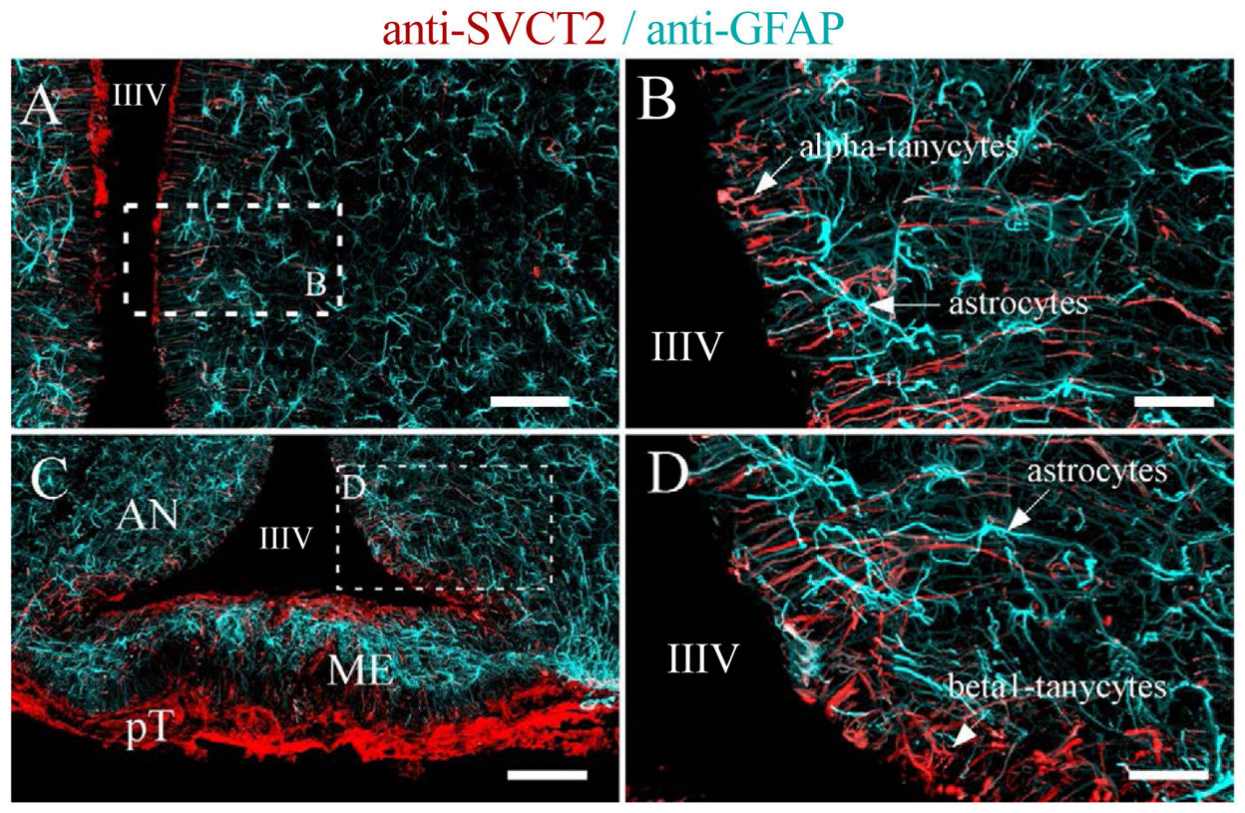
SVCT2 is highly expressed in hypothalamic tanycytes Tanycytes and astrocytes are specialized glial cells distributed in the hypothalamus. Tanycytes can be classified as alpha (A–B) or beta (C–D), which contact the CSF of the third ventricle (apical area); the processes contact different areas of the hypothalamic region (basal area). Astrocytes are present in the hypothalamic sub-ependymal region and are concentrated in the median eminence (C, light-blue staining) below the ventricular layer (beta-2 tanycytes). SVCT2 and GFAP immunofluorescence analysis was performed using confocal spectral microscopy (Zeiss 780 equipment), tile scanning and Z-stack projection imaging. An intense immunoreaction for SVCT2 was detected in alpha and beta tanycytes (red signal); however, astrocytes, endothelial cells, and neurons were negative for SVCT2 staining. Astrocytes showed an intense immunoreaction for GFAP (light-blue staining). IIIV, Third ventricle; AN, Arcuate nucleus; GFAP, glial fibrillary acidic protein; ME, Median eminence; pT, Pars tuberalis. Scale bars in A and C, 500 μm; in B and D, 40 μm.

AA concentrations vary within the brain; higher AA concentrations are consistently observed in the hippocampus and hypothalamus compared with other structures within the CNS [69]. In the hypothalamus, vitamin C modulates nitric oxide neurotransmission [70]. Tanycytes are specialized hypothalamic glial cells localized in circumventricular organs, such as the median eminence (Figure 3) [71–74]. Tanycytes are classified into at least four types, alpha1, alpha 2, beta1, and beta 2, and immunofluorescence analyses revealed the strongest immunoreaction for SVCT2 in β1 and β2 tanycytes [65]. Ultrastructural immunohistochemistry confirmed that SVCT2 was localized in the cellular membranes of the apical microvilli and blebs of β1 tanycytes, and AA transport by SVCT2 within these cells was confirmed using primary cultures of tanycytes, demonstrating that AA transport within these cells is Na+ dependent and unaffected by CytB, a GLUT inhibitor [65]. In this report, we used spectral confocal microscopy, z-stack projection and 3D rendering analysis to confirm SVCT2 expression in alpha and beta hypothalamic tanycytes (Figure 3A and 3G), which also showed an intense immunoreaction for vimentin (Figure 3B–3D and 3F). These cells were also identified using an adenovirus expressing enhanced green fluorescence protein (EGFP; Figure 3E). SVCT2 was not detected in hypothalamic astrocytes, which presented high GFAP expression (Figure 4A–D); its expression was also absent in endothelial cells and hypothalamic neurons (Figure 4). SVCT2 expression in tanycytes and the high concentration of AA in the hypothalamus suggest a neuroprotective mechanism for concentrating vitamin C in this specific area of the brain that is in contact with the CSF and blood vessels.

Along with neurons and hypothalamic glial cells, immunohistochemistry analyses revealed SVCT2 expression in microglia, ependymal cells and choroid plexus cells [31,57]. Double immunohistochemistry analysis demonstrated SVCT2 co-localization with OX42, a microglia cell marker [57]. However, this data contrasts with *in situ* hybridization data that did not find SVCT2 expression in oligodendrocytes and microglia due to the absence of SVCT2 labeling in white matter regions of the brain [64].

## Vitamin C Recycling and the Bystander Effect

The oxidized form of vitamin C, DHA, can be incorporated into cells by the facilitative glucose transporters, GLUTs, because DHA shares a similar structure with glucose [5,75]. Because the transport of DHA can occur in either direction, the cell must trap the vitamin C by converting DHA to its reduced form, AA. The capacity to take up DHA and reduce it to AA has been observed in endothelial cells, muscle cells, chondrocytes, and cells of the hemato-retinal barrier [76–79].

Many cell types can incorporate vitamin C principally through GLUTs while DHA concentrations remain extremely low [80]. For example, human neutrophils accumulate intracellular vitamin C at concentrations up to a millimolar range during an oxidative burst [81]. Also, melanoma tumor cells can increase the capture of DHA up to 10-fold; however, a similar increase in AA uptake was not observed [82]. Altered expression of certain GLUT isoforms was also observed in these cells [83]. The absolute specificity of these cells for DHA is in apparent contradiction to evidence indicating that vitamin C is present in human blood only as AA; however, melanoma cells have strong pro-oxidant activity and may be able to generate substantial concentrations of DHA outside the cell [84].

For normal and tumor cells, a mechanism has been proposed to describe how they capture vitamin C through GLUTs in the presence of cells in an oxidative state: the bystander effect. Superoxide generated by activated cells undergoing oxidative burst converts extracellular AA to DHA, which is then transported through GLUTs by the activated cells as well as other cells present in the immediate area, including those that are unable to either transport AA or produce superoxide [30]. The intracellular DHA is then immediately reduced back to AA, which accumulates at high intracellular concentrations. This theory is supported by recent studies of co-cultures of adherent bystander cells (DU-145 human prostate cancer cells, breast cancer cell lines, melanoma cell line, human umbilical vein endothelial cells) with PMA-activated cells (HL-60 neutrophils and normal human neutrophils) in which the uptake of vitamin C by bystander cells was inhibited by CytB, a known inhibitor of GLUTs [30,85]. The uptake was also independent of sodium, demonstrating that the bystander cells captured vitamin C in the form of DHA through GLUTs [30,85]. Because of the ubiquitous expression of GLUTs, all cells have the potential capacity to acquire vitamin C through the bystander mechanism.

## Recycling of Vitamin C within the Brain

**Figure 5:**
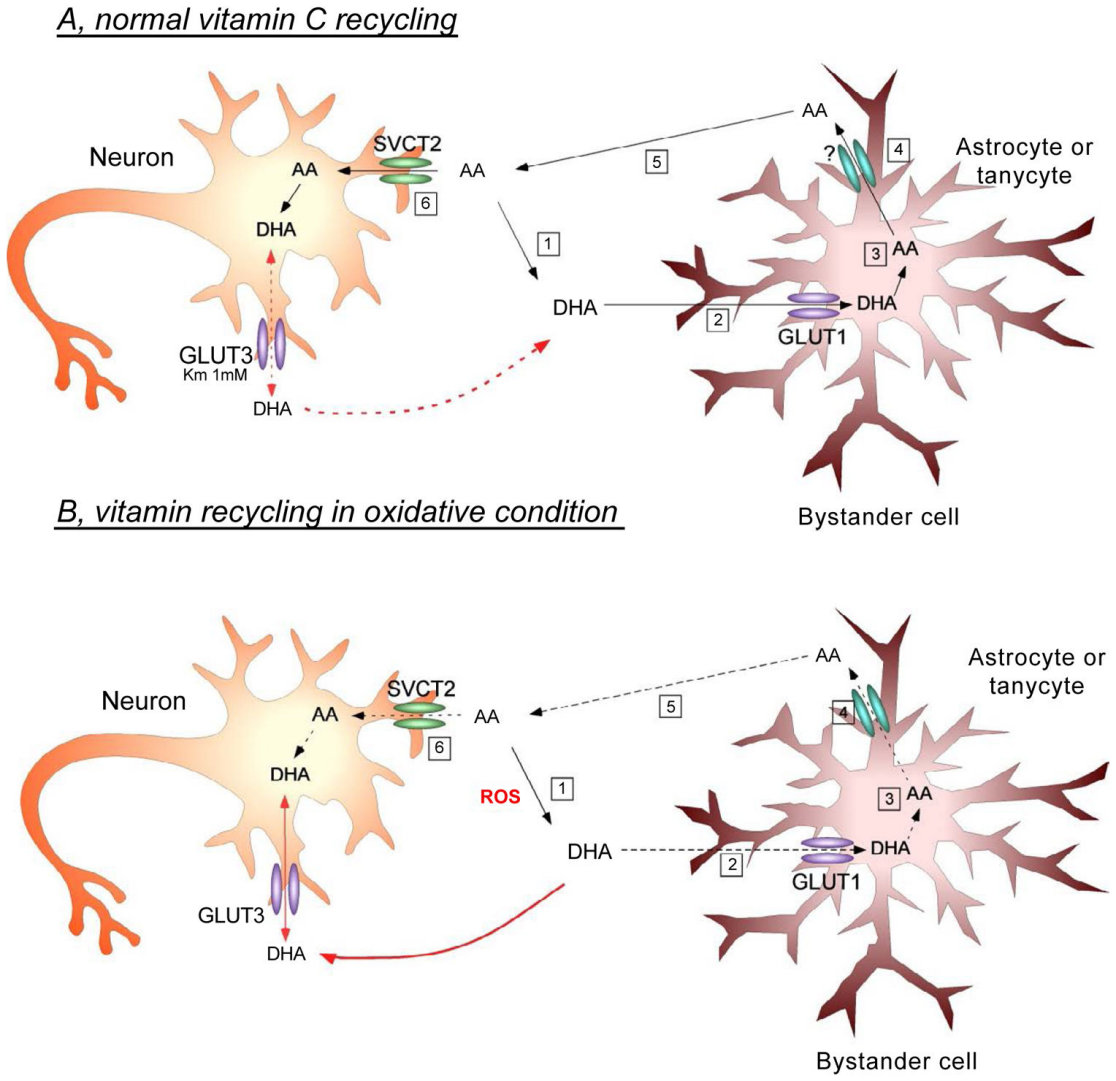
Vitamin C recycling and proposed interaction between neurons and astrocytes in normal or oxidative stress conditions of the brain. A Under physiological conditions, reactive oxygen species (ROS) are constantly generated in the CNS. In these conditions, ROS oxidizes AA to DHA (A1), which preferentially enters the astrocyte by GLUT1 (A2). Inside the astrocyte, DHA is again reduced to AA (A3), and is then released into the extracellular space by a yet unknown mechanism (A4). The extracellular AA then enters the neuron via SVCT2 (A5/6), exerting its antioxidant effect and thereby protecting neurons against cell death. Moreover, under normal conditions, we postulate that GLUT3 expression by neurons is involved mainly in DHA efflux. Increased intracellular DHA concentration in the neuron inhibits glycolysis, increases PPP activity, consumes glutathione and increases the influx of lactate. This adaptive mechanism changes the normal metabolism of the neurons in response to the accumulation of DHA [19]. **B.** In pathophysiological conditions, ROS is massively generated (B1), resulting in increased extracellular DHA concentrations and subsequent DHA entry into astrocytes. However, given the large amount of oxidizing species, the astrocyte will not be able to efficiently reduce DHA to AA (B3), resulting in less AA efflux into the extracellular environment (B4). Subsequently, AA enters the neuron through SVCT2 (B5/6), bringing about a decrease in intracellular antioxidant protection due to its lower degree of uptake by the neuron. Simultaneously, the large extracellular generation of DHA promotes its entry into neurons via GLUT3 (red arrow). Finally, the massive incorporation of intracellular DHA may promote neuronal death by a yet unknown mechanism.

The bystander effect model can also describe vitamin C recycling between astrocytes and neurons in which neurons are the “activated cells” that induce oxidation of AA to DHA, which is subsequently taken up by the “bystander cell,” astrocytes. Dehydroascorbic reductase may play a pivotal role in regenerating AA from its oxidized product; however, this enzyme has only been localized in large neurons (i.e. pyramidal neurons and Purkinje neurons) [86]. Alternatively, neuronal intracellular AA may be oxidized to generate DHA, which may exit neurons through facilitated diffusion and enter astrocytes via GLUTs. Inside astrocytes DHA is reduced back to AA, which may be released through neurotransmitter stimulation, anion channels or glutamate heteroexchange [62] and taken up again by neurons via SVCT2 (Figure 5A).

### Entrance of DHA into astrocytes and DHA conversion to AA

GLUT 1 and GLUT3 are the isoforms most common in the brain and were the first to be detected [21,87,88]. GLUT1 is the primary isoform expressed in astrocytes while neurons primarily express GLUT3 [89–91]. As discussed previously, SVCT2 is not expressed in astrocytes thus limiting the influx of vitamin C in astrocytes to GLUTs [31,62,65]. We have shown that primary cultures of astrocytes after 7 days *in vitro* may concentrate vitamin C via the expression of GLUT1 [21]. These cells only uptake DHA and not AA at ample levels, and this uptake is unaffected by the lack of Na+, but is greatly inhibited by CytB, a GLUT inhibitor [20].

Because the transport of DHA can occur in either direction, the cell must convert DHA to its reduced form, AA, to maintain intracellular vitamin C concentrations. The capacity to take up DHA and the later reduce it to AA has been observed in endothelial cells, muscle cells, chondrocytes, and cells of the hemato-retinal barrier [76–79]. The mechanism by which astrocytes reduce DHA to AA remains to be elucidated; however, a potential mechanism involving glutathione and other reducing enzymes has been proposed for the interconversion of oxidized and reduced forms of vitamin C [92,93]. Astrocytes have four times more glutathione than neurons, and potentially a higher capacity to reduce vitamin C and the deleterious effects of DHA [62]. Thus in this model, extracellular DHA enters astrocytes through GLUT1 and is immediately converted to AA (Figure 5A).

### Efflux of AA from astrocytes

After the conversion of DHA to AA within astrocytes, AA efflux is necessary for its uptake by neurons and other cells within the brain (Figure 5A). The possible mechanisms that have been proposed for the efflux of AA from astrocytes include neurotransmitter stimulation, anion channels or glutamate heteroexchange. For example, primary cultures of rat astrocytes increased AA release under hypotonic conditions, which was inhibited by anion-transport inhibitors, DIDS and 4,4′-dinitrostildene-2,2′-disulfonic acid (DNDS), thus suggesting an anion channel-regulated efflux of AA from astrocytes under swelling conditions [94]. A glutamate-ascorbate heteroexchange mechanism was first proposed after observing that increased glutamate uptake by brain cells also induced AA efflux [16,95–98]; this was also confirmed in astrocytes [99]. However, this theory is controversial due to the fact that hypertonic medium as well as volume-sensitive osmolyte and anion channel (VSOAC) inhibitors prevented the efflux, and Ca+ influx was also observed [100]. These data suggest that either volume or calcium-mediated anion channel mechanisms could induce AA efflux.

### Entrance of AA into neurons and conversion to DHA

After the release of AA from astrocytes (Figure 3), extracellular AA is capable of entering neurons via SVCT2 [56]. Interestingly, although neurons express a plentiful amount of GLUT3, transport of glucose was inhibited after AA accumulation in both cortical and hippocampal neurons *in vitro* while astrocytes were unaffected [21]. The inhibition of glucose transport was also accompanied by increased lactate transport [101]. Thus, AA enters neurons and can serve as an antioxidant or metabolic modulator. Neurons exhibit a much higher level of oxidative metabolism then other cells, which would convert AA back to DHA [102]. DHA efflux has been observed in neurons, which most likely prevents the toxic effects of DHA [103]; this efflux may likely occur by facilitated transport via GLUT3. Extracellular DHA may then return to astrocytes via GLUT1 to be converted again to AA thus completing the circuit (Figure 5A).

## SVCT2 Expression and Recycling under Abnormal Conditions

To further understand the role and transport of vitamin C in the brain, SVCT2 expression has also been studied under abnormal condition (oxidative stress). No changes in SVCT2 expression by astrocytes were observed in the penumbra of an excitotoxic lesion [64]. However, after middle cerebral artery occlusion, SVCT2 expression pattern was altered; its expression was lost in the ischemic core, which correlated with the general loss of protein synthesis [104]. Outside the ischemic core, SVCT2 expression increased in the penumbral and peri-intact regions. Furthermore, SVCT2 expression was increased in neurons as well as astrocytes [104], which may explain how astrocytes in culture develop SVCT2 expression due to exposure to constant oxidative stress. It is also interesting to note that SVCT2 expression in microglial cells or oligodendrocytes was not observed, demonstrating that these cells may never gain the capacity to express SVCT2 even under oxidative stress [104].

Therefore, an alternative cycle to the one presented above is possible (Figure 5B). Under oxidative conditions, released AA from astrocytes would be converted to DHA by extracellular oxidants. DHA could then enter into astrocytes or neurons via GLUT1 or GLUT3, respectively, and possibly mediate increased cellular toxicity, at least in neurons. Additionally, astrocytes may increase the expression of SVCT2, incorporating a higher level of AA that is normally available only to neurons.

## Conclusions

SVCT2 is the primary vitamin C transporter expressed in neurons and some specialized cells, including microglia, tanycytes, choroid plexus and stem cells, of the CNS. In response to oxidative stress, astrocytes also express SVCT2. Although blood-brain-barrier endothelial cells do not express SVCT2, choroid plexus cells of the blood-CSF barrier are positive for SVCT2 expression, suggesting that this transporter also plays an essential role in the entrance of vitamin C into the brain. During periods of low plasma AA levels, the brain and CSF are among the last tissues to lose their ascorbate. Conversely, during times of high circulating levels of ascorbate, the concentration within the brain and CSF does not dramatically change, suggesting the presence of an AA recycling mechanism within the brain that involves DHA uptake by astrocytes through GLUTs, its conversion to AA, release from the cell, and entry into neurons through SVCT2. Recent studies describing the transport and recycling of vitamin C into and within the brain through the *bystander effect* have illustrated the unique mechanism by which the CNS maintains constant levels of vitamin C in normal conditions and its influence during pathological conditions. Increased ROS levels in the CNS could perturb AA recycling, increasing oxidative damage due to DHA accumulation in neurons. This condition can be a general state that enhances the early development of various pathologies of the nervous system that directly or indirectly cause permanent oxidative damage.
